# The Structure and the Function of the Cochlear Intra-Strial Fluid-Blood Barrier

**DOI:** 10.4172/2161-119x.1000308

**Published:** 2017-05-22

**Authors:** Xiaohan Wang

**Affiliations:** Department of Otolaryngology/Head and Neck Surgery, Oregon Hearing Research Center, Oregon Health and Science University, USA

## Introduction

The fluid-blood barrier selectively excludes most blood-borne substances from entering the inner ear, protecting tissues from factors in the blood which would perturb the homeostasis [1]. In her paper, Dr. Shi timely reviews current views on the structure and function, pathology and therapeutic targets of the blood barrier in the stria vascularis. The blood barrier in the stria vascularis is a specialized capillary networks in the cochlea, which also constitutes part of the blood-labyrinth barrier (BLB) in the inner ear.

The primary physical diffusion barrier of the blood barrier in the stria vascularis consists of endothelial cells (ECs) connected together by tight junctions and adherens junctions. In addition to ECs, the BLB includes mural cells such as pericyte (PC) (about 1220–1330 PCs populated on capillaries in stria vascularis of a normal adult C57BL/6J mouse) [2]. PCs support the abluminal capillary wall and are embedded in the basement membrane (BM). PCs expressing platelet derived growth factor receptor-β (PDGFR-β), desmin, neural/glial antigen (NG2) and CD90, play an important role in vascular integrity, angiogenesis and BM formation. In addition to the EC and PC which are building blocks of capillaries, an interstitial cell type in the stria vascularis, named as perivascular resident macrophage type-melanocytes (PVM/Ms), situates closely to capillaries and displays the characteristics of both macrophage and melanocyte. PVM/Ms express the typical macrophage surface markers F4/80, CD68, CD11b and scavenger receptor classes A (1) and B (1), as well as the melanocyte surface markers glutathione S-transferase nalpha4 and Kir4.1 [3,4]. ECs, PCs and PVM/Ms together form a functional “cochlear vascular unit” in the stria vascularis. Interaction between the three cell types is critical for controlling vascular integrity, vascular permeability, providing a suitable microenvironment for hearing function.

There is currently little information on the regulation of permeability in the stria vascularis. Upregulation of tight junctions and adherens junction proteins decreases permeability and strengthens the barrier, while down-regulation of the proteins increases permeability [2]. In addition to the exchange by para-cellular pathways, substances are also exchanged by transcellular pathways. Near 40% proteins in the barrier are involved in transport activities.

Dysfunction of the blood barrier in the stria vascularis is implicated in noise-induced hearing loss (NIHL) [5], age-related hearing loss [6], autoimmune diseases [7], genetic hearing disorders [8] and inflammatory conditions in the inner ear [9]. In NIHL, increased vascular permeability, ischemia, infiltration of leukocytes and endothelial injury are frequently observed. Activation of PVM/Ms may contribute to the increased vascular permeability by reducing the production of pigment epithelium-derived factor, leading to down-regulation of tight junction-associated proteins. In age-related hearing loss, capillary damage and degeneration are quite evident. Thickened basement membrane, a reduced number of PCs and morphological and functional changes in PVM/Ms may induce dysfunction in the blood barrier, leading to hearing loss. In autoimmune disease, there is strong evidence that the blood barrier in the stria vascularis is targeted by immune system. For example, autoimmune antibodies or circulating immune complexes were found to lead to barrier breakdown and hearing defects. Genetic defects in BLB components have been identified in multiple genetic hearing disorders including Norrie disease, Alport disease, Nr3b2 (−/−), light (Blt), white spotting (Ws) and Varitint-Waddler-J (Vaj) mouse mutants and connexin deficiency related hearing loss. In inflammation induced hearing disorder, vascular integrity is compromised in the blood barrier in the stria vascularis. Lipopolysaccharide (LPS) disrupts the blood barrier in the stria vascularis by down regulating tight junction proteins, which is shown to increase entry of serum fluorescein into the perilymph through the strial blood barrier. The blood barrier also is the target of ototoxic drugs such as aminoglycoside, cisplatin, carboplatin and furosemide [10,11]. These drugs are taken up by transporter systems in the strial endothelium.

This review also includes discussion of the experimental models currently available to study blood barrier permeability control and properties. In particular, 2D *in vitro* cell line based-models which include a co-cultivated Transwell model, conditioned medium co-culture model and CytoVu/SiMPore thin membrane co-culture model are very useful for studying cell-cell interaction in control of barrier integrity [2]. On the other hand, a newly developed 3D model matrigel matrix is ideal for studying how the interactions of individual vascular cells give rise to vessel properties [12]. Several *in vivo* models are recently available which include intravital florescence microscopy (IVM) [13] and enhanced magnetic resonance imaging (MRI) technique [14,15]. In particular, IVM approach is used in conjunction with an open vessel-window to directly observe blood vessel activities. MRI techniques provide valuable information on structures, functions and metabolic states in intact cochleae. Dynamic contrast enhanced (DCE) MRI using gadolidium-chelate (GdC) or gadolidium based contrast agents (GBCA) is especially useful for assessing vascular permeability.

In summary, this new review provides a comprehensive synopsis of the structure and function intra-strial fluid-blood barrier under physiological and pathological conditions.
